# Supplementary material for: Morphology and surface chemistry engineering toward pH-universal catalysts for hydrogen evolution at high current density
Source: Nat Commun. 2019 Jan 17;10:269. doi: 10.1038/s41467-018-07792-9 (PMC6336864; doi:10.1038/s41467-018-07792-9)
Supplement: Supplementary file 1 — Supplementary information [file 41467_2018_7792_MOESM1_ESM.pdf]

Morphology and Surface Chemistry Engineering for pH-Universal Catalysts toward Hydrogen Evolution at Large Current Density

Yuting Luo[1], Lei Tang[1], Usman Khan[1], Qiangmin Yu[1], Hui-Ming Cheng[1, 2], Xiaolong Zou[1*] & Bilu Liu[1*]

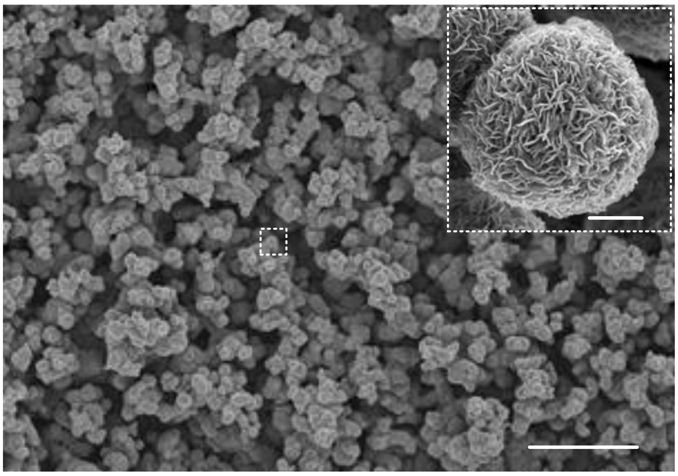

Supplementary Figure 1. SEM image of the synthesized MoS_2 microspheres. The porous structure is composed of microspheres of radially-aligned MoS_2 nanosheets with an average thickness of 10 nm. The scale bar is 20 μm. Inset shows an enlarged SEM image of a typical MoS_2 microsphere. The scale bar is 500 nm.

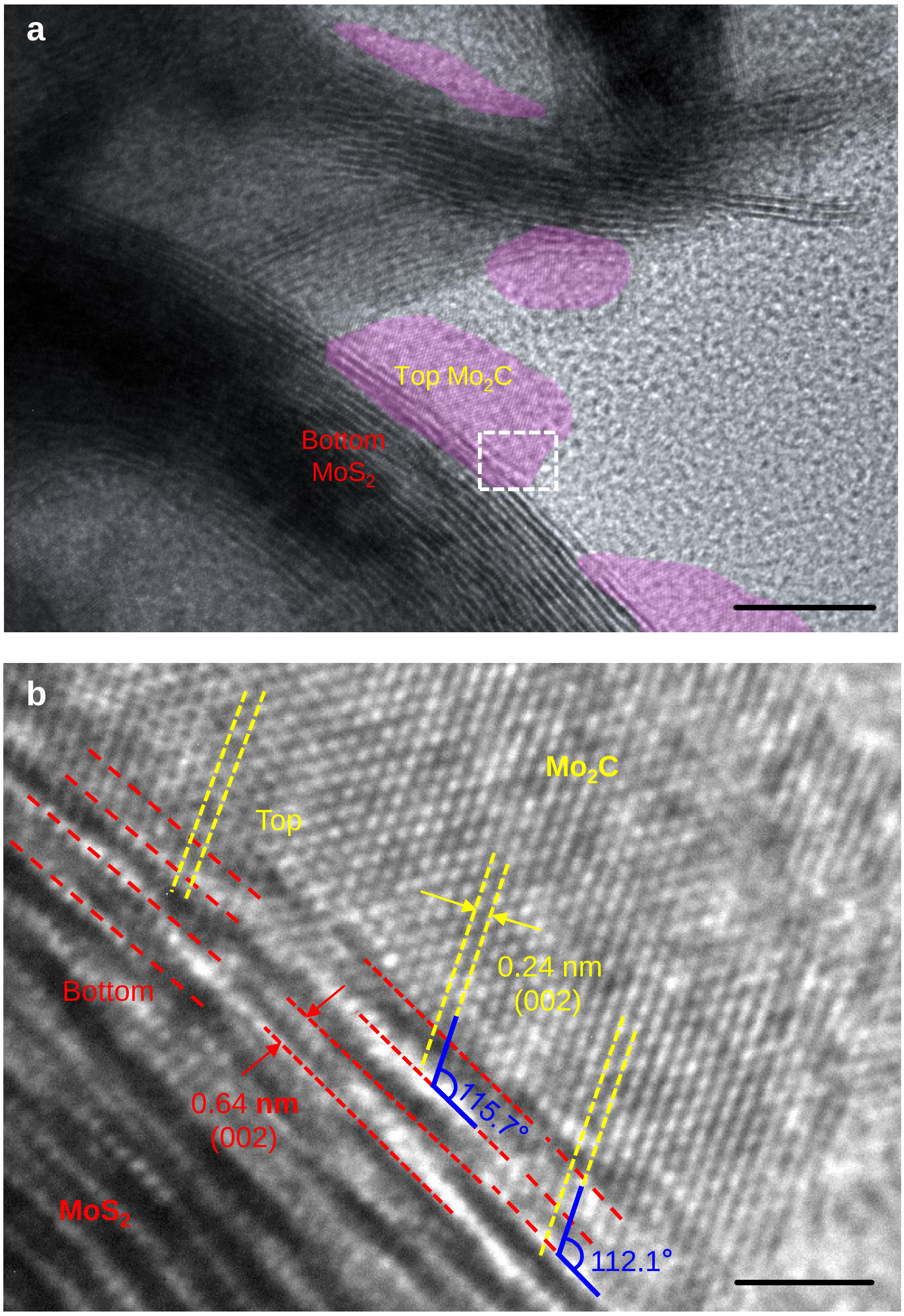

Supplementary Figure 2. HRTEM images of the structure and interface of Mo_2C and MoS_2 in MoS_2/Mo_2C. a The Mo_2C nanoparticles are highlighted by the pink areas. The results show that β-phase Mo_2C nanoparticles have grown at the edges of MoS_2 nanosheets. The scale bar is 10 nm. **b** An enlarged view of the dotted rectangular area in a. The scale bar is 2 nm.

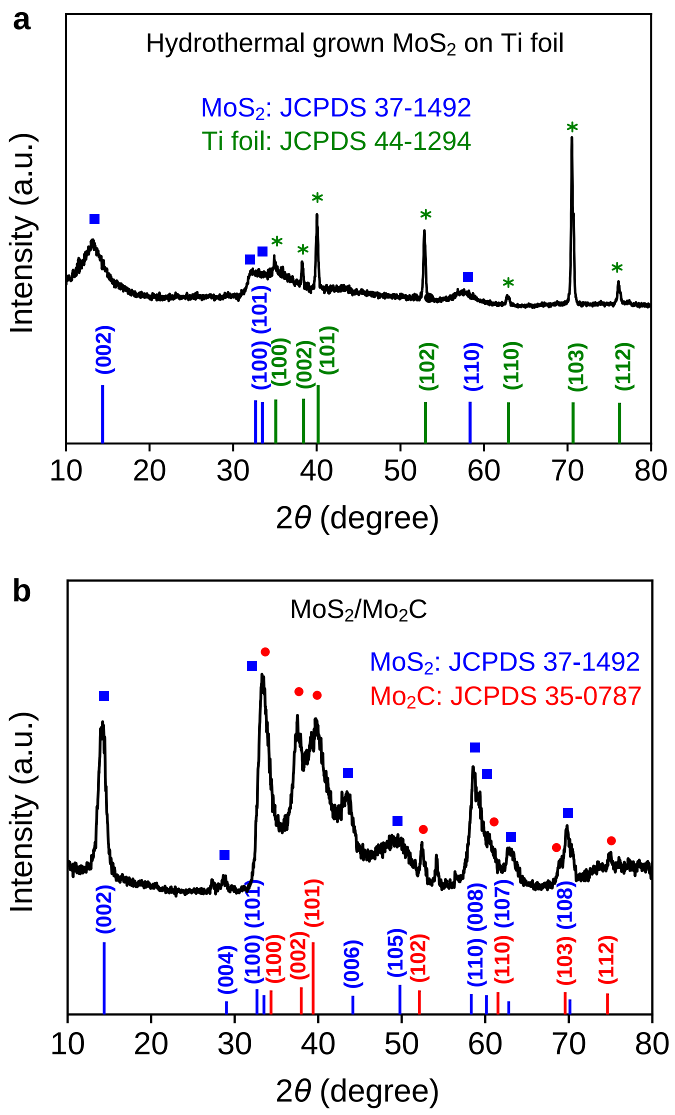

Supplementary Figure 3. XRD patterns of as-synthesized materials on Ti foils. a XRD pattern of the MoS_2. **b** XRD pattern of the MoS_2/Mo_2C. The results show that after high temperature CVD, β-Mo_2C was formed and the crystallinity of the MoS_2 improved. This may be the reason why signals from Ti substrate disappeared in b.

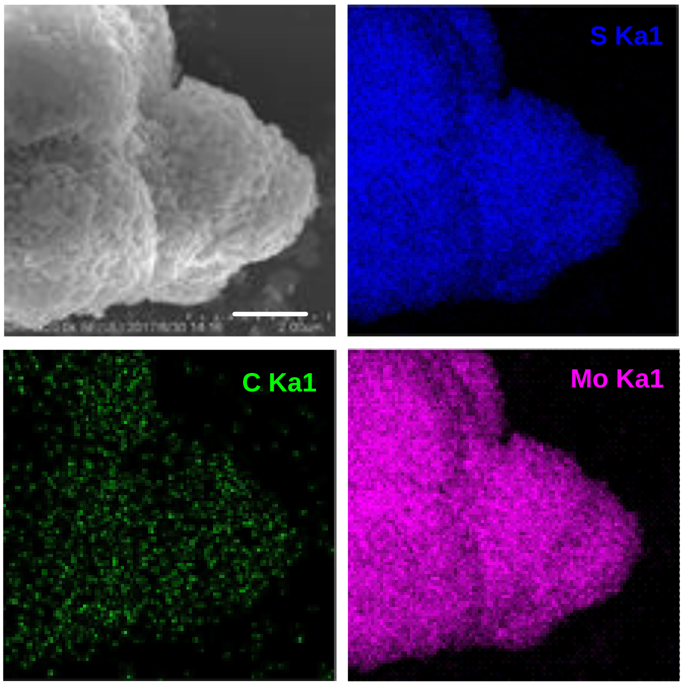

Supplementary Figure 4. EDS elemental maps of MoS_2/Mo_2C. The results showing the uniform distributions of S, C, and Mo in the MoS_2/Mo_2C samples.

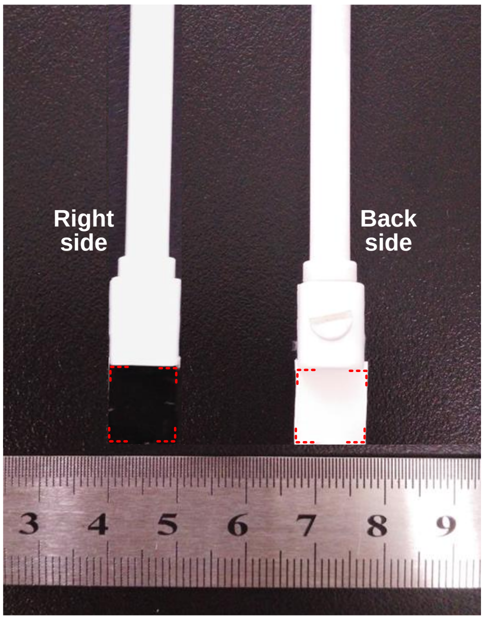

Supplementary Figure 5. Optical image showing the front and back sides of a working electrode with the back side been covered by insulating tape. The front side (black) is MoS_2 or MoS_2/Mo_2C. The tape covering ensures an identical size of exposed surface area for the different samples.

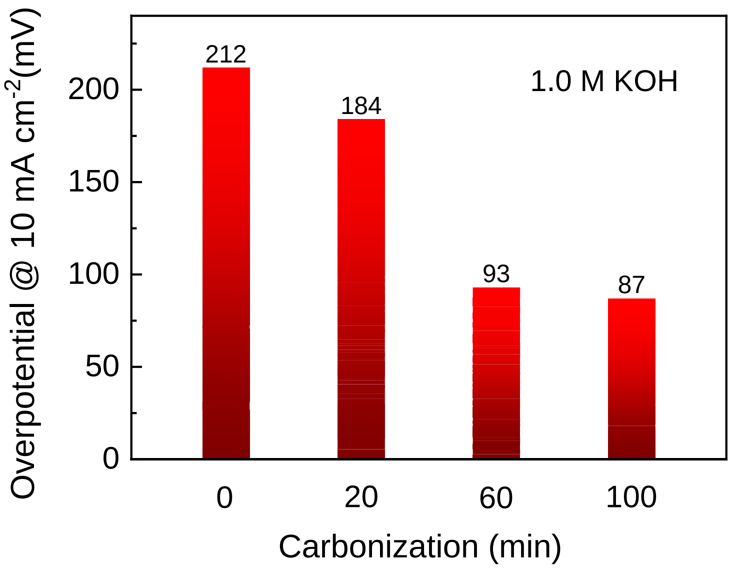

Supplementary Figure 6. Overpotentials at 10 mA cm^{-2} for MoS_2 microspheres after different carbonization times. The electrochemical measurements were performed in a 1.0 M KOH electrolyte. The results show that the overpotentials of MoS_2 decrease rapidly as the carbonization time increases from 0 min to 60 min, and later becomes relatively stable with a further increase in time from 60 min to 100 min. In our experiments, we used MoS_2 samples with 60 min carbonization.

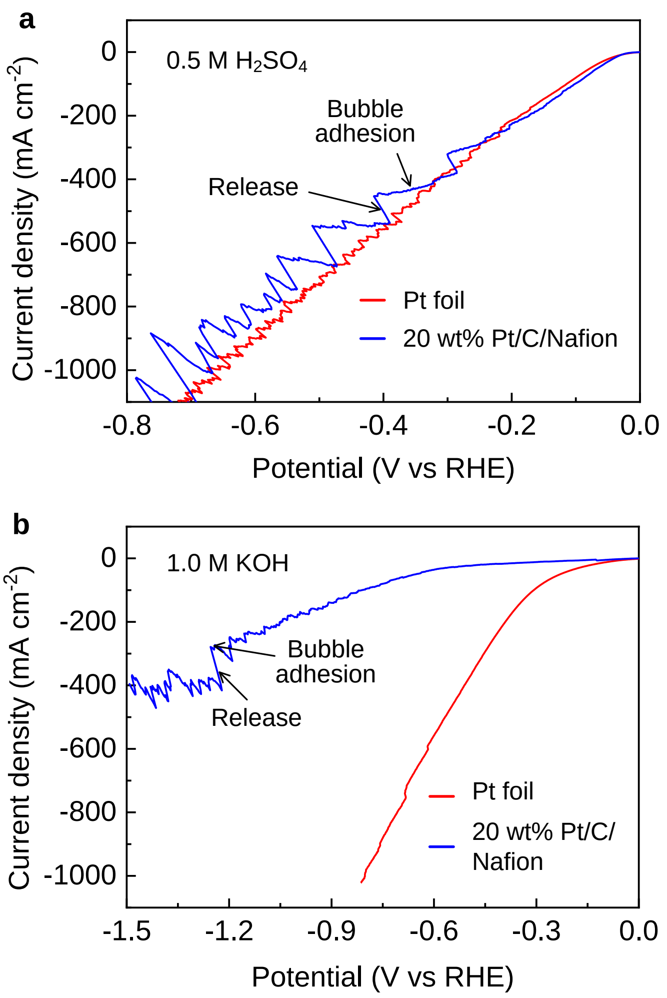

Supplementary Figure 7. Polarization curves for a Pt foil and 20 wt% Pt/C/Nafion. **a** 0.5 M H_2SO_4 electrolytes. **b** 1.0 M KOH electrolytes. The scan rate is 5 mV s^{-1}. The results show that Pt foils show comparable or even better HER performance than Pt/C/Nafion at high current densities. To prepare the Pt/C/Nafion electrode, Pt/C powder (2 mg) was dispersed in a mixture of water/ethanol (4:1 vol) by sonication. Then, a certain amount of the dispersion was drop cast onto a glassy carbon electrode with a diameter of 3 mm. After this, 2 μL of a 5 wt% Nafion solution was coated on the Pt/C and dried in order to avoid the Pt/C catalyst peeling off at high current densities. Pt/C supported on the glassy carbon electrode was put into the solution without rotation or stirring so as to make a fair comparison with the other samples and its electrochemical properties studied.

Supplementary Table 1. A comparison of the HER activity of Pt/C and Pt foils in alkaline media obtained in this work compared with those reported in the literature.

Catalysts	Loading amount (μg cm^{-2})	Electrolyte	Current density (mA cm^{-2})	Overpotential η (mV)	Reference
20 wt% Pt/C on Ni foam	2000	1.0 M KOH	10<br>100<br>1000	~30<br>~90<br>N.A	1
20 wt% Pt/C on Ni foam[2]	1000	1.0 M KOH	10<br>100<br>1000	~35<br>~110<br>N.A	2
20 wt% Pt/C	143	1.0 M KOH	10<br>100<br>1000	~25<br>~200<br>N.A	3
20 wt% Pt/C	120	1.0 M KOH	10<br>100<br>1000	~40<br>>250<br>N.A	4
Pt metal	4500	1.0 M KOH	10<br>100<br>1000	~100<br>~250<br>N.A	4
**Pt foil**	**--**	**1.0 M KOH**	**10<br>100<br>1000**	**~60<br>~300<br>~800**	**This work**
**20 wt% Pt/C**	**200**	**1.0 M KOH**	**10<br>100<br>1000**	**~45<br>~250<br>N.A**	**This work**
**20 wt% Pt/C/Nafion**	**200**	**1.0 M KOH**	**10<br>100<br>1000**	**~30<br>~780<br>>900**	**This work**

Supplementary Table 2. A comparison of the HER activity of Pt/C and Pt foils in acidic media obtained in this work compared to those reported in the literature.

Catalysts	Loading amount (μg cm^{-2})	Electrolyte	Current density (mA cm^{-2})	Overpotential η (mV)	Reference
20 wt% Pt/C	360	0.5 M H_2SO_4	10<br>100<br>1000	~30<br>~100<br>N.A	5
20 wt% Pt/C	~400	0.5 M H_2SO_4	10	~50	6
40 wt% Pt/C	193	0.5 M H_2SO_4	10<br>100<br>1000	45<br>180<br>1120	7
20 wt% Pt/C	360	0.5 M H_2SO_4	10<br>100	30<br>97	5
20 wt% Pt/C	200	0.5 M H_2SO_4	10<br>100	25<br>90	8
20 wt% Pt/C	700	0.5 M H_2SO_4	10<br>100<br>1000	29<br>122<br>948	9
20 wt% Pt/C	400	0.5 M H_2SO_4	10<br>100<br>200	~20<br>~70<br>~105	10
**Pt foil**	**--**	**0.5 M H_2SO_4**	**10<br>100<br>1000**	**~30<br>~115<br>~660**	**This work**
**20 wt% Pt/C**	**200**	**0.5 M H_2SO_4**	**10<br>100<br>1000**	**~30<br>~105<br>N.A**	**This work**
**20 wt% Pt/C/Nafion**	**200**	**0.5 M H_2SO_4**	**10<br>100<br>1000**	**~30<br>~115<br>~670**	**This work**

As can be seen from Supplementary Tables 1 and 2, our work shows a comparable HER performance of the Pt catalysts with that reported in recent papers. Note that, compared to the 20 wt% Pt/C/Nafion electrode, the 20 wt% Pt/C electrode shows better HER activity in alkaline media at a large current density because the Nafion film covering the Pt/C may decrease the mobility of hydroxyl ions in

alkaline media. The 20 wt% Pt/C electrode was prepared by dispersing Pt/C powder (2 mg) in a water/ethanol/Nafion solution (32/7/1 vol), and subsequently dropping a certain amount of the dispersion onto a glassy carbon electrode. However, the Pt/C films prepared by this method are not firm enough and peel from the carbon glass electrode as current density increases to 1000 mA cm^{-2}.

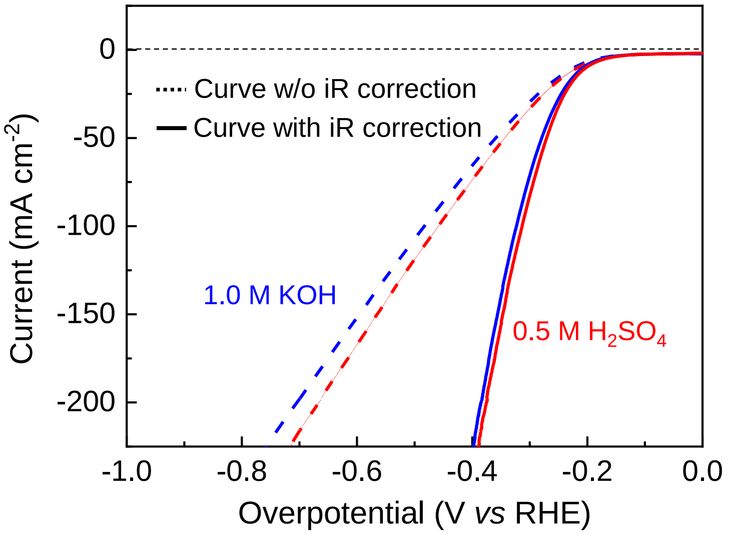

Supplementary Figure 8. Polarization curves of MoS_2–H samples in KOH (1.0 M) and H_2SO_4 (0.5 M). The result shows that the HER performance of MoS_2-H is a little better than pristine MoS_2, but much worse than MoS_2/Mo_2C, indicating Mo_2C plays an important role in the good HER performance of MoS_2/Mo_2C.

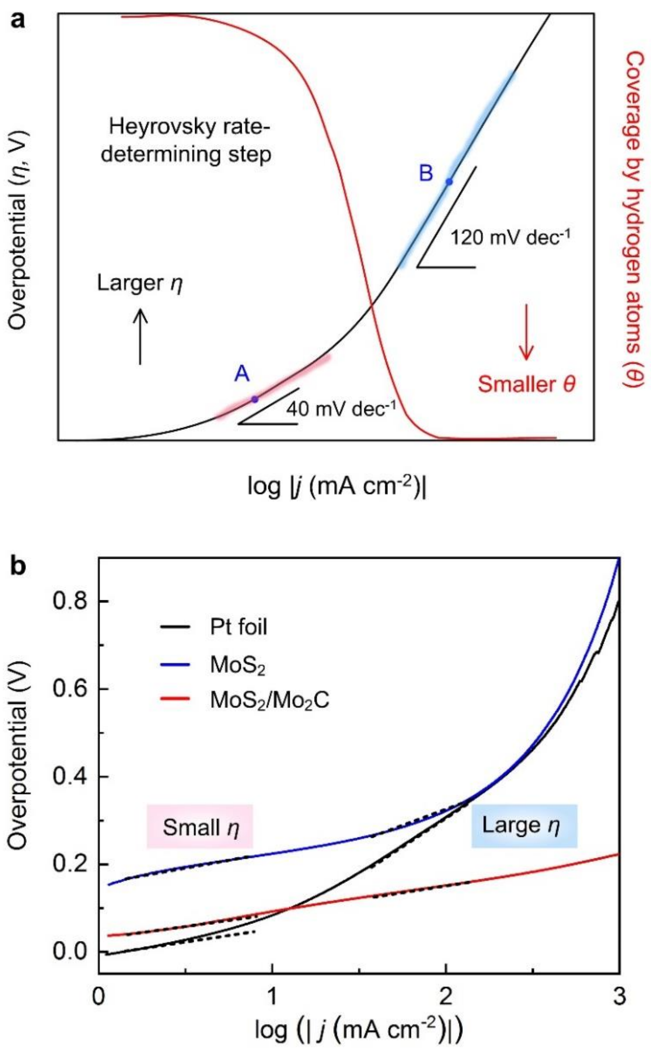

Supplementary Figure 9. Theoretical potential-dependent Tafel relationship for HER if the Heyrovsky step rate-determining. a Schematic showing that if the overpotential (η) is small (corresponding to a large surface coverage of hydrogen atoms (θ)), the Tafel slope is close to 40 mV dec^{-1}, exemplified by point A. As η increases (corresponding to θ decreases), the Tafel slope increases and reaches 120 mV dec^{-1}, exemplified by point B. Therefore, there is a potential-dependent, and in turn, a θ dependent Tafel relationship.[11] **b** Experimental HER results show that the Pt foil and MoS_2 have potential-dependent Tafel relationships, while the MoS_2/Mo_2C has a small slope (*i.e.*, the current density increases a lot as overpotential increasing) even at a large current density of 1000 mA cm^{-2}. The electrolyte is 1 M KOH.

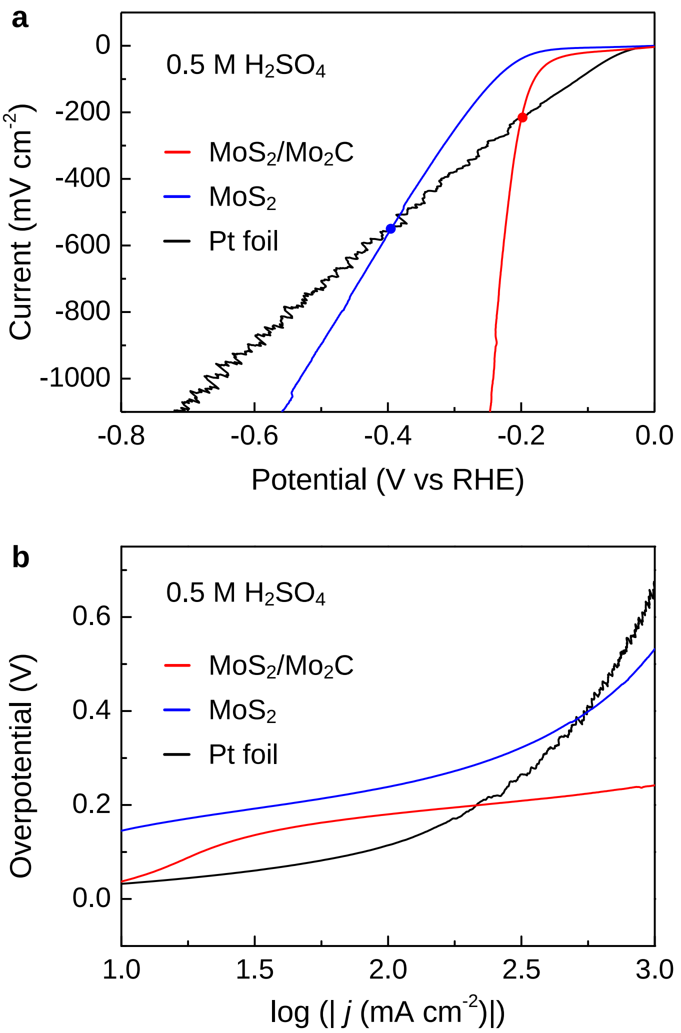

Supplementary Figure 10. HER performance of MoS_2/Mo_2C in 0.5 M H_2SO_4. a Polarization curves for MoS_2/Mo_2C, MoS_2, and a Pt foil. **b** Tafel curves for MoS_2/Mo_2C, MoS_2, and a Pt foil. The scan rate is 5 mV s^{-1}. The results show that in acidic media, the three samples have a similar trend to that in alkaline media, *i.e.*, as current density increases, MoS_2/Mo_2C maintains a small Tafel slope (close to 40 mV dec^{-1}) while the Pt foil and MoS_2 show increasing Tafel slopes (much larger than 40 mV dec^{-1}).

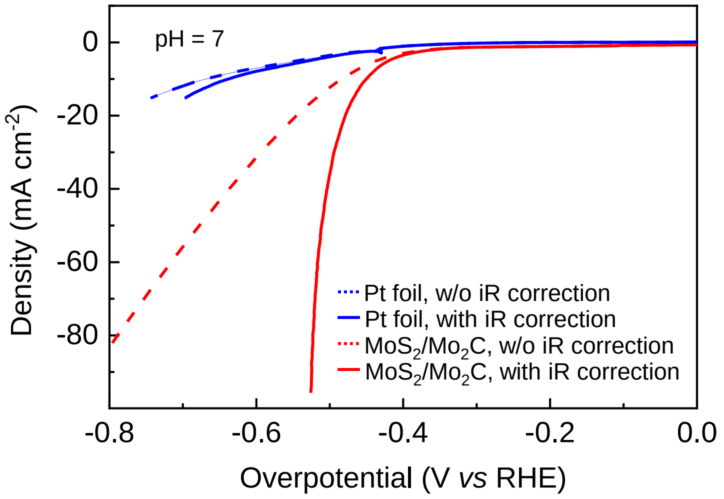

Supplementary Figure 11. Polarization curves with and without iR correction for a Pt foil and a MoS_2/Mo_2C sample in a neutral condition. The electrolyte is 0.5 M Na_2SO_4 and the scan rate is 5 mV s^{-1}. The MoS_2/Mo_2C shows a smaller overpotential (449 mV) than Pt foil (636 mV) at 10 mA cm^{-2}.

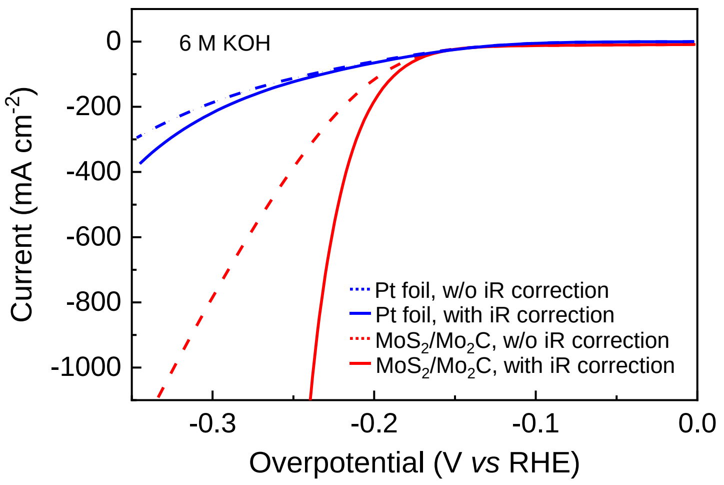

Supplementary Figure 12. **Polarization curves with and without iR correction for Pt foil and MoS_2/Mo_2C in concentrated KOH (6 M) solutions.** The scan rate is 5 mV s^{-1}.

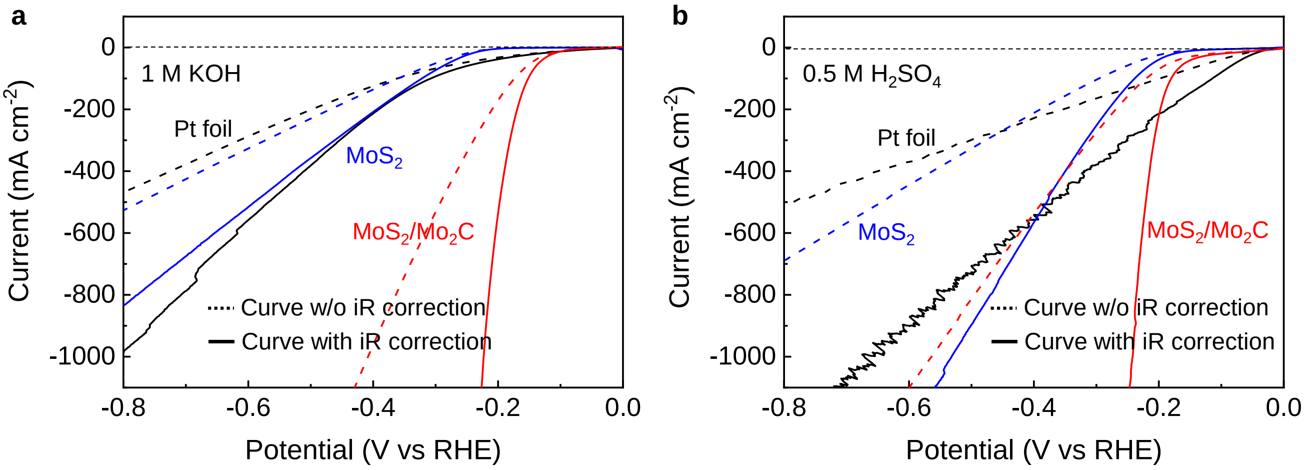

Supplementary Figure 13. **Polarization curves for MoS_2/Mo_2C, MoS_2, and a Pt foil. a** 1.0 M KOH electrolytes. **b** 0.5 M H_2SO_4 electrolytes. The dotted lines denote raw data without iR correction, while the solid lines denote those with iR correction.

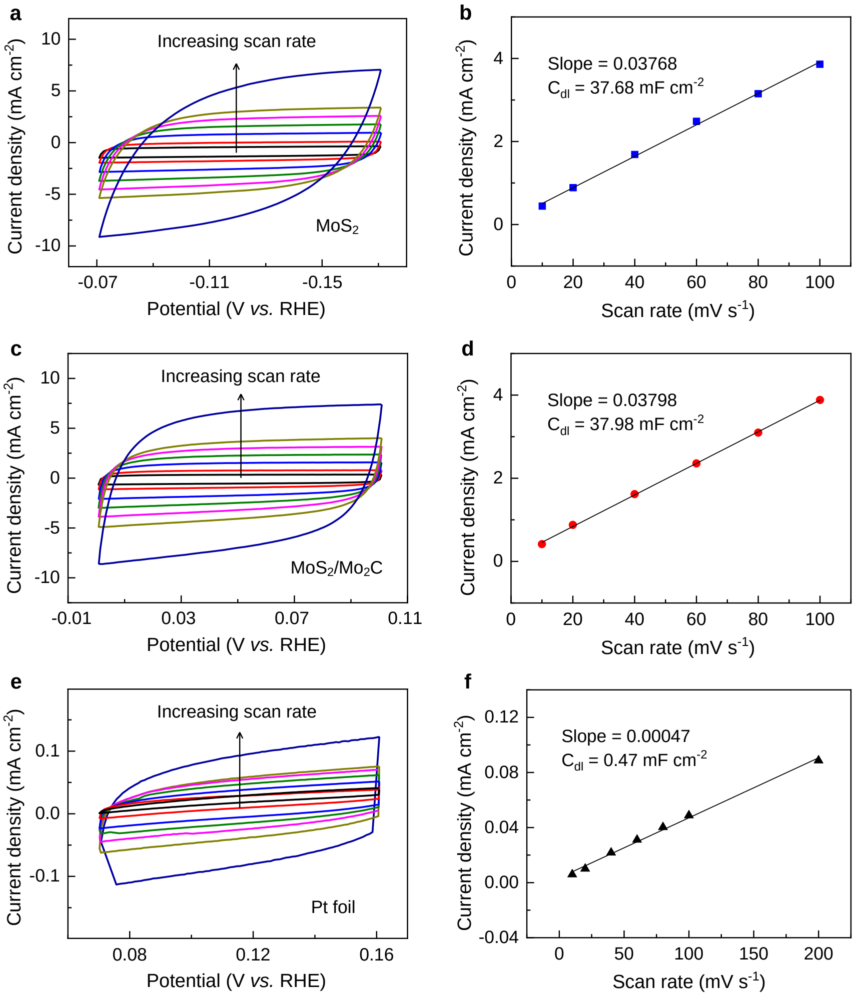

Supplementary Figure 14. **CV curves at different scan rates and the C_{dl} of MoS_2, MoS_2/Mo_2C, and Pt foil. a, c, e** CV curves at different scan rates. **b, d, f** CV and capacitive current plotted as a function of scan rate in 1.0 M KOH at scan rates of 10, 20, 40, 60, 80, 100, and 200 mV s^{-1} for (a, b) MoS_2, (c, d) MoS_2/Mo_2C, and (e, f) Pt foil. The results show that the C_{dl} values for the MoS_2 and MoS_2/Mo_2C, which are proportional to the electrochemical surface area, are very close (37.68 and 37.98 mF cm^{-2}), suggesting similar numbers of catalytically active sites in the two electrocatalysts for HER. The C_{dl} of Pt foil (0.47 mF cm^{-2}) is much less than MoS_2 and MoS_2/Mo_2C, suggesting better intrinsic per-site activity of Pt than MoS_2 and MoS_2/Mo_2C. The better intrinsic activity of Pt leads to better HER activity of Pt foil at relatively low current density of 10 mA cm^{-2} than the other two catalysts.

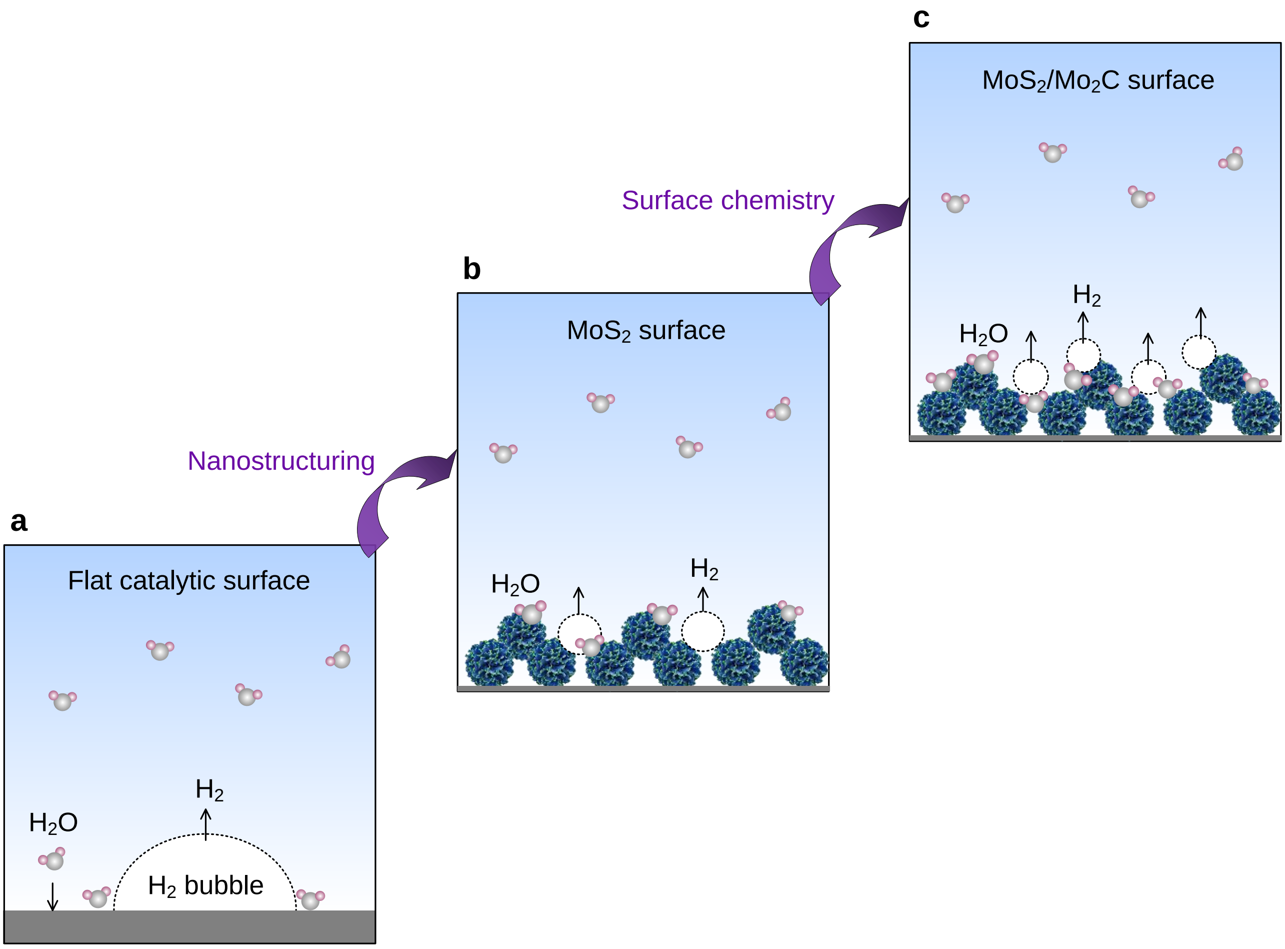

Supplementary Figure 15. Schematic showing that combined surface chemistry and morphology of MoS_2/Mo_2C may improve the transfer of reactants and hydrogen bubbles. a Flat catalytic surface with poor wettability. **b** MoS_2 surface. **c** MoS_2/Mo_2C surface. Compared to flat catalytic surface with poor wettability and MoS_2 surface, reactants are more accessible to MoS_2/Mo_2C surface, and it is easier for the small hydrogen gas bubbles generated to escape from the MoS_2/Mo_2C surface and uncover a fresh surface for subsequent reactions.

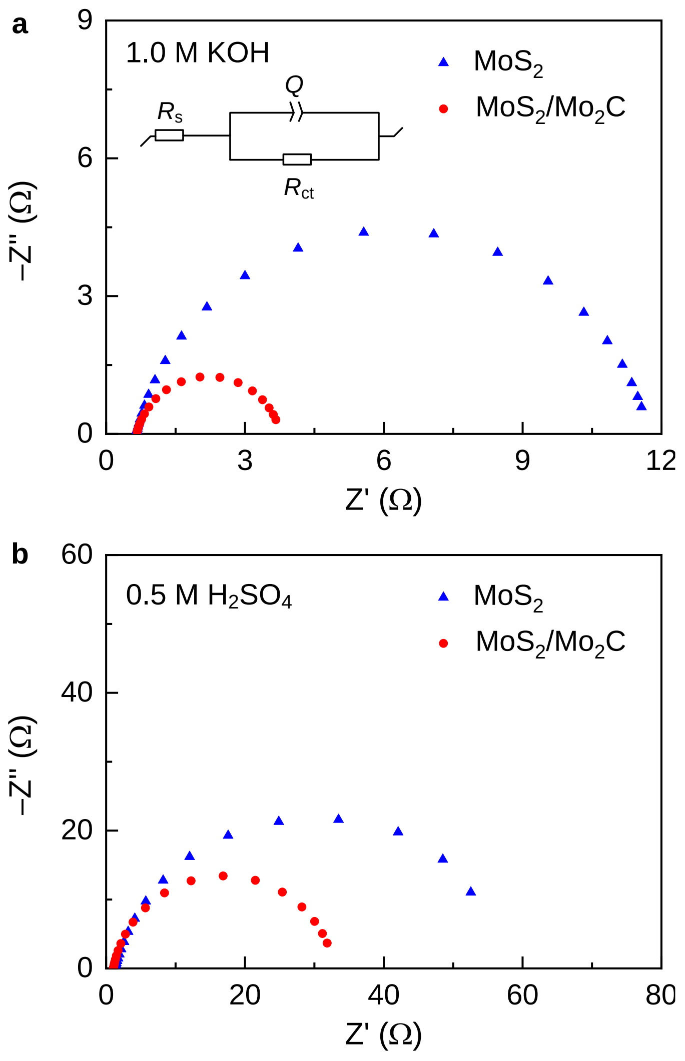

Supplementary Figure 16. **EIS curves of MoS_2/Mo_2C and MoS_2**. **a** 1.0 M KOH electrolytes. **b** 0.5 M H_2SO_4 electrolytes. The Nyquist plots show that the MoS_2/Mo_2C has better electron transfer ability than the MoS_2 in both media, further confirming the good HER kinetics of the MoS_2/Mo_2C. The inset in Supplementary Figure 16a illustrates the equivalent circuit of the system.

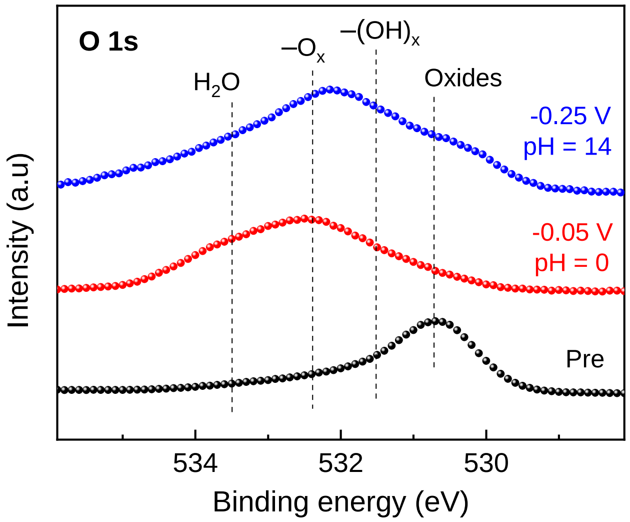

Supplementary Figure 17. O 1s XPS spectra of β-Mo_2C that first underwent the HER tests and were then directly transferred to XPS chamber for measurements using a vacuum transfer stage. Note that the samples were not exposed to atmosphere during whole process. The results show very similar oxygen peaks to those shown in Figure 4a in the main text.

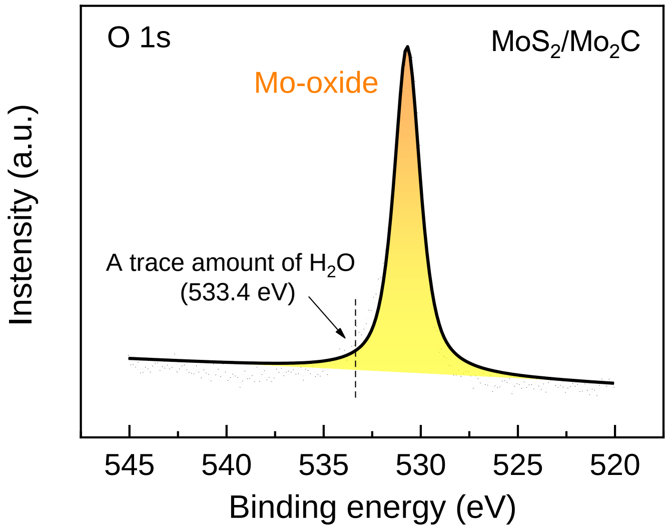

Supplementary Figure 18. **O 1s XPS spectrum of a MoS_2/Mo_2C sample stored in a glovebox for 12 hrs immediately after the carbonization.** The results show that only a main peak at 530.8 eV appears, indicating that there is no organic contamination during 12 hrs storage in glove box.

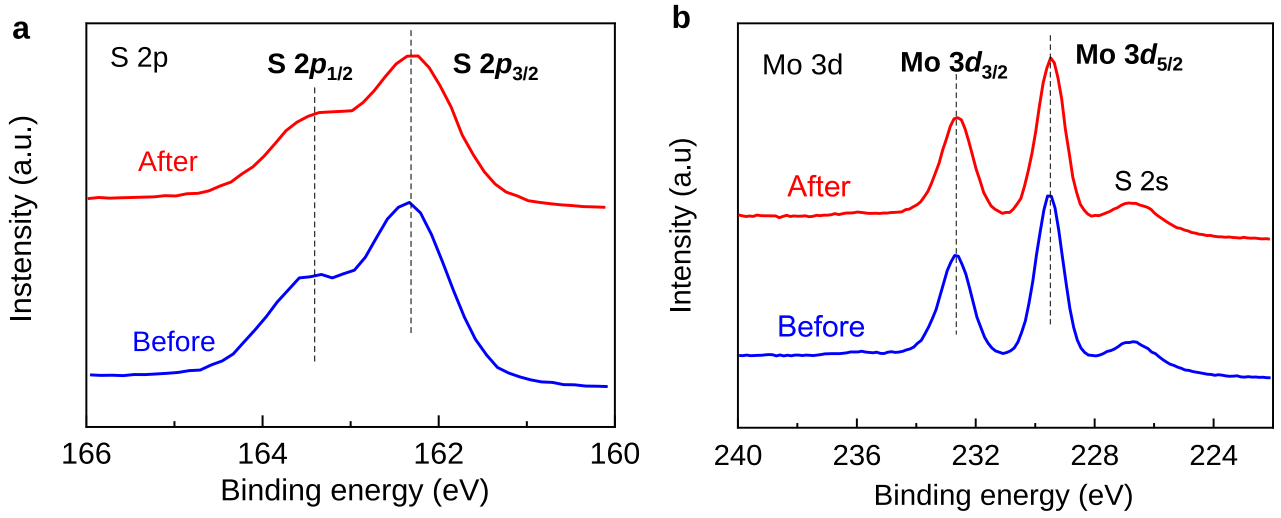

Supplementary Figure 19. **XPS spectra of MoS_2 before and after HER cycling. a** S 2p spectra. **b** Mo 3d XPS spectra. The results showing negligible changes of the binding energies and surface chemistry of MoS_2.

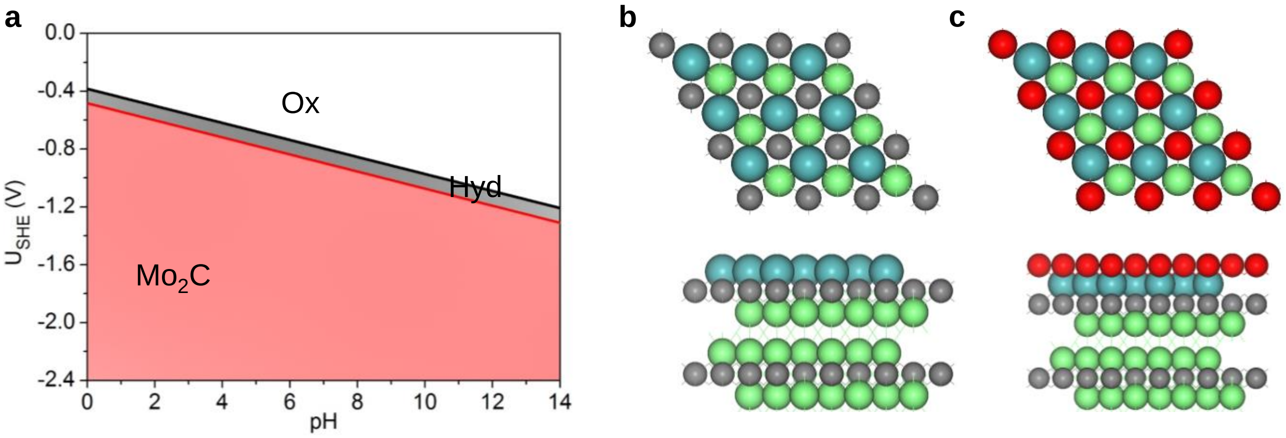

Supplementary Figure 20. **DFT calculations. a** Pourdaix diagram of Mo_2C (001) surface in different pH and voltage conditions. **b, c** Hydrogen and water absorption on the Mo_2C (001) surface. The results show that the (001) surface of Mo_2C is also modified by surface oxygen species during HER. However, the water dissociation kinetics and hydrogen absorption/deposition kinetics on the (001) surface are not as good as on the (101) surface, leading to differences in their HER activities. Here, the (dark) green, grey, red, and white spheres represent Mo, C, O, and H atoms, respectively.

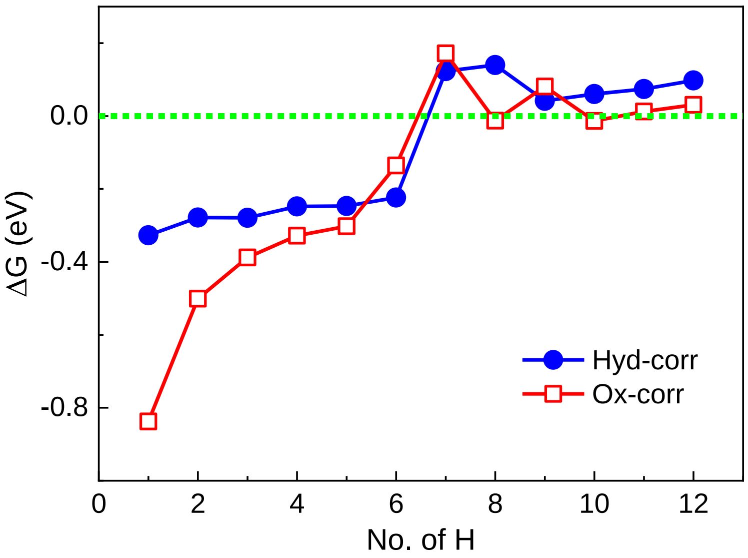

Supplementary Figure 21. DFT calculated adsorption energies for H on –O (Ox) or –OH (Hyd) terminated Mo_2C (101) surfaces. Ox-corr and Hyd-corr stand for the adsorption energies corrected by a shift of 0.1 eV for PBE functional. The results show that H adsorption energies on –O terminated Mo_2C (101) surface are closer to 0 than those on –OH terminated surface at high hydrogen coverage, suggesting higher performance of MoS_2/Mo_2C in alkaline media than in acidic media.

Using RPBE functional, the optimal H absorption energy of ~ -0.09 eV on Pt(111) is obtained for 0.25 ML coverage.[12] It has been pointed out by Bandarenka *et al.*[13] that the adsorption energies of H on Pt using PBE functional are stronger than those using RPBE functional, and increasing H coverage will improve agreement between them. Accordingly, we take our results of H adsorption energy with 1ML coverage on Pt(111) using PBE functional for comparison. The H adsorption energy is ~ -0.18 eV, which is ~0.1 eV stronger than that using RPBE functional.

Supplementary References

1 Chen, Y.-Y. *et al.* Self-Templated Fabrication of $MoNi_4/MoO_{3-X}$ Nanorod Arrays with Dual Active Components for Highly Efficient Hydrogen Evolution. *Adv. Mater.* **29**, 1703311, (2017).

2 Zhang, J. *et al.* Efficient Hydrogen Production on Moni4 Electrocatalysts with Fast Water Dissociation Kinetics. *Nat. Commun.* **8**, 15437, (2017).

3 Lin, H. *et al.* Cobalt-Doping in Molybdenum-Carbide Nanowires toward Efficient Electrocatalytic Hydrogen Evolution. *Adv. Funct. Mater.* **26**, 5590-5598, (2016).

4 Feng, J.-X., Tong, S.-Y., Tong, Y.-X. & Li, G.-R. Pt-Like Hydrogen Evolution Electrocatalysis on Pani/Cop Hybrid Nanowires by Weakening the Shackles of Hydrogen Ions on the Surfaces of Catalysts. *J. Am. Chem. Soc.* **140**, 5118-5126, (2018).

5 Xing, Z., Liu, Q., Asiri, A. M. & Sun, X. Closely Interconnected Network of Molybdenum Phosphide Nanoparticles: A Highly Efficient Electrocatalyst for Generating Hydrogen from Water. *Adv. Mater.* **26**, 5702-5707, (2014).

6 Wang, D. Y. *et al.* Highly Active and Stable Hybrid Catalyst of Cobalt-Doped FeS_2 Nanosheets-Carbon Nanotubes for Hydrogen Evolution Reaction. *J. Am. Chem. Soc.* **137**, 1587-1592, (2015).

7 Zhu, L. *et al.* A Rhodium/Silicon Co-Electrocatalyst Design Concept to Surpass Platinum Hydrogen Evolution Activity at High Overpotentials. *Nat. Commun.* **7**, 12272, (2016).

8 Yin, J. *et al.* Ni-C-N Nanosheets as Catalyst for Hydrogen Evolution Reaction. *J. Am. Chem. Soc.* **138**, 14546-14549, (2016).

9 Chen, Y. *et al.* Highly Active, Nonprecious Electrocatalyst Comprising Borophene Subunits for the Hydrogen Evolution Reaction. *J. Am. Chem. Soc.* **139**, 12370-12373, (2017).

10 Han, N. *et al.* Nitrogen-Doped Tungsten Carbide Nanoarray as an Efficient Bifunctional Electrocatalyst for Water Splitting in Acid. *Nat. Commun.* **9**, 924, (2018).

11 Shinagawa, T., Garcia-Esparza, A. T. & Takanabe, K. Insight on Tafel Slopes from a Microkinetic Analysis of Aqueous Electrocatalysis for Energy Conversion. *Sci. Rep.* **5**, 13801, (2015).

12 Norskov, J. K., *et al*. Trends in the Exchange Current for Hydrogen Evolution. *J Electrochem. Soc.* **152**, J23–J26 (2005).

13 Marcus, D. P., Sebastian, W., Federico, C.-V. & Aliaksandr S. B. Nature of Highly Active Electrocatalytic Sites for the Hydrogen Evolution Reaction at Pt Electrodes in Acidic Media. *ACS Omega*, **2**, 8141–8147 (2017).
