# Supplementary material for: Morphology and surface chemistry engineering toward pH-universal catalysts for hydrogen evolution at high current density
Source: Nat Commun. 2019 Jan 17;10:269. doi: 10.1038/s41467-018-07792-9 (PMC6336864; doi:10.1038/s41467-018-07792-9)
Supplement: Supplementary file 3 — Description of Additional Supplementary Information [file 41467_2018_7792_MOESM3_ESM.pdf]

Description of Additional Supplementary Files

File Name: Supplementary Movie 1
Description: Hydrogen bubble behaviors on the flat Pt and MoS_2/Mo_2C sample surfaces, indicating that the structures with roughness at both the micro- and nanoscale could accelerate the release of hydrogen bubbles.

File Name: Supplementary Movie 2
Description: Hydrogen bubble behaviors on the MoS_2 and MoS_2/Mo_2C sample surfaces, indicating the importance of interactions at the atomic level (that is the surface chemistry) to the release of hydrogen bubbles.
